# Spatial and temporal assessment of snake encounters in urban Delhi, India

**DOI:** 10.1038/s41598-023-50373-0

**Published:** 2024-03-06

**Authors:** Gaurav Barhadiya, Jayaditya Purkayastha, Ashis Kumar Saha, Chirashree Ghosh

**Affiliations:** 1 Ramanujan College, University of Delhi, New Delhi, Delhi 110019 India; 2 Help Earth, Guwahati, Assam 781007 India; 3 Department of Geography, University of Delhi, New Delhi, Delhi 110007 India; 4 Department of Environmental Studies, University of Delhi, New Delhi, Delhi 110007 India

**Keywords:** Ecology, Ecology, Environmental sciences

## Abstract

Delhi, the capital city of India is, highly urbanized and surrounded by remnant forest, farms, ridges, and other green areas experience regular snake encounters in and around residential, institutional, and industrial areas. A total of 41 months of sampling from January 2019 to May 2022 was conducted wherein we, studied the snake assemblage in Delhi to determine the species composition, encounter frequency, seasonal activity patterns, and probable encounter sites in an urban setup. We documented 372 individuals belonging to 15 species from seven families out of 23 species found in Delhi. Snakes were found inside forests, public parks, homes, drain networks, streets, office buildings, and even in school-college buildings. The most recorded species being *Ptyas mucosa* (37.37%, n = 139), *Naja naja* (19.62%, n = 73), and *Lycodon aulicus* (13.44%, n = 50). The highest numbers of incidents were reported in the month of July (22.04%, n = 82) and August (19.89%, n = 74) during the peak monsoon season, for identifying high encounter sites, we used a geostatistical modeling tool, Ordinary kriging to identify places having more snake occurrences. We further used a statistical spatial method called average nearest neighbor distance to detect the pattern distribution of snake species. Spatial interpolation done through Ordinary kriging highlighted two areas having concentrated snake encounters. The results of the average nearest neighbor distance analysis showed three species having clustered and two species having dispersed distribution. The incidence of snake encounters was found to be highly seasonal and appeared to be associated mainly with monthly rainfall, temperature, and humidity. The findings of this study on snakes’ distribution patterns provide valuable insights into the conservation of these species. Understanding their habitat preferences and spatial distribution is crucial for the implementation of effective conservation strategies.

## Introduction

Cities have now been recognized as biodiversity hotspots^1^ as they are providing habitat for a wide range of flora and fauna^2^. Cities provide habitat by the means of mosaics of ‘ecological niches’ which are generally formed by urban trees, public parks, private gardens, lawns, urban forests, wetlands, and streams^3^. Wildlife is known to be attracted to urban setups due to the ecosystem services associated with synanthorpization, which often results in increased rates of human-wildlife conflicts^4^. Among them, accidental encounters with snakes in urban areas are a major challenge, which ultimately results in the increased competition between snakes and humans for resources like food and space^5^. Unfortunately, very less is known about the impact of anthropogenic disturbance on reptiles, especially snakes within the urban matrix^6^.

To date very few studies have been carried out on snakes in an urban environment, even when carried out, most studies focus on protected areas like national parks or wildlife sanctuaries and ignore the major portion of the city’s urban matrix^7^. In India, some studies^8–11^ have been carried out on snake assemblage inhabiting an urban area, out of published work, articles mainly mentioned species found in the area and didn’t address the distribution or ecology of snakes inhabiting urban areas.

Delhi, the national capital territory of India is the second most populated city in the world and is known for its greenery with around 22% green cover^12^. It is surrounded by agricultural farms, ridges, and other green areas and experiences regular snake occurrences in residential, recreational, educational, and industrial areas^10^. Unfortunately, snakes in Delhi have never received priority as compared to the other groups like mammals and avian fauna, therefore, no significant studies have been published on the occurrence of snakes in Delhi^13^.

Populations of the animal show a wide variety of distribution patterns that are not easy to describe precisely, once distribution patterns are found it is important to identify what are the factors responsible for such patterns and what is the mechanism responsible for the same^14^. In this study, we recorded information on snake encounters over a period of 41 months (January 2019 to May 2022) to understand the species composition, encounter frequency, seasonal activity patterns, and probable encounter sites, using geostatistical modeling and statistical tools in highly urbanized Delhi. Finally, we identified the regions having a high frequency of snake encounters.

## Materials and methods

### Study area

**Figure 1:**
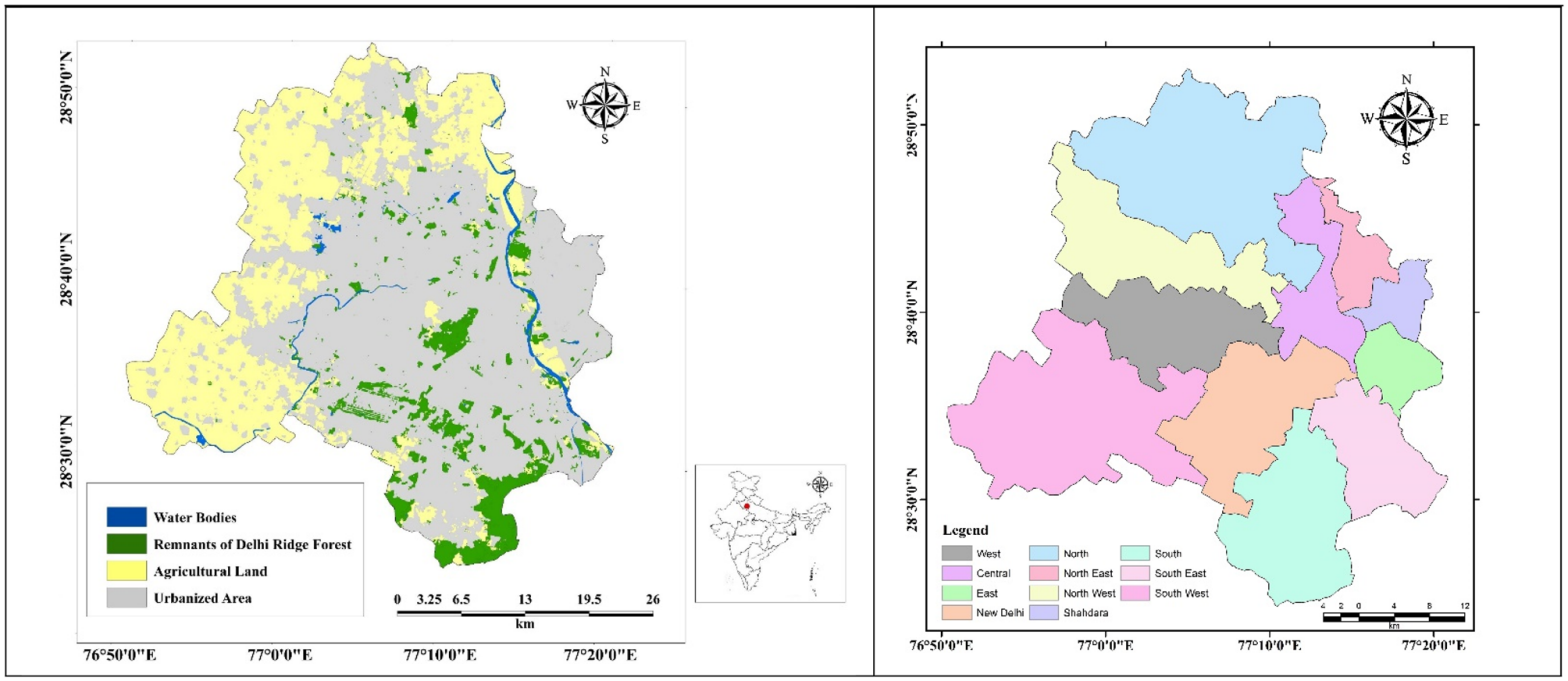
Study area, Union territory of Delhi, highlighting the Urbanized areas in grey, remnants of Delhi ridge forest in green, Agricultural lands in yellow, and river and water bodies in blue (Left) and Eleven administrative Districts (Right). *Source* Left: India: Sentinel-2 10 m Land Use/Land Cover 2021 (https://livingatlas.esri.in/server/rest/services/Imagery/IN_S2_LULC_2021/MapServer)*. Right: NCT of Delhi: Education (https://livingatlas.esri.in/server/rest/services/Delhi/DL_Education/MapServer/0)**. Composed using ESRI ArcGIS 10.2 Software. *This work is licensed under a Creative Commons by Attribution (CC BY 4.0) license. **ESRI, India.

Delhi (Fig. 1) is among the most populous cities in the world with a population of 16.7 million as on March 2011 with an annual average growth rate of 1.92%. The overall population density is 11,297 km^2^^12^. The study area, situated between 28.24° to 28.53° N and 76.50° to 77.20° E, experiences temperature variations from 7 ± 3 °C in winter to 45 ± 3 °C in summer^15^. It is located at an elevation of 216 m above sea level and covers an area of approximately 1482 km^2^^16^.

93% population in the city is living in urban areas as compared to the national average of 31.16%^12^. It has a semi-arid climate and the city is surrounded by the mountain region of the Himalayas to the north, the central hot peninsular region to the south, the hilly region to the east, and, to the west the Great Indian Desert^17–20^. Delhi gets most of its rainfall during the monsoon season and the total average rainfall received is 611.8 mm/year^21^. Two prominent features of Delhi’s geography are the Yamuna River and the Delhi Ridge located within the city boundary. The Ridge is part of the Aravalli Mountain which is around 1800 million years old. The city has 299.7 km^2^ of forest Cover, which is 20.2% of the total geographical area of the city^22^, Delhi has a typical Northern tropical thorn forest type^23^, that includes exotic *Prosopis juliflora* (commonly known as Mesquite), *Acacia nilotica* (Babool), *Acacia leucophloea* (Ronjh), *Acacia catechu* (Khair), *Butea monosperma* (Dhak), *Cassia fistula* (Amaltas), *Salvadora persica* (Meswak) and *Anogeissus latifolia* (Dhau) are the common trees available in abundant^22^.

### Data collection

Snakes were recorded in Delhi through active searches, attending to rescue calls, opportunistic encounters, and archives of the snake rescue organizations of the Union territory of Delhi from January 2019 to May 2022 Eight men hour were invested per survey employing Visual Encounter Survey^24^.

Sixty four men hours were invested per month, survey including Diurnal and nocturnal (two times a day for two hours each i.e. 8 am to 10 am and 8 pm to 10 pm) active searches for snakes were conducted twice a week (eight times a month) at different forests and in urban parks. Systematic active searches were conducted in various wastelands and within the diverse built-up environment of urban Delhi, which is a probable habitat for snakes, where snakes had been previously encountered or reported. These searches spanned from January 2019 to May 2022 to determine the historical observations of snakes. We also informed the general public about our research through personal level communication and through various social networking sites. We did not encourage people to actively search for or capture snakes. Instead, we distributed our phone numbers to various local community leaders and presented our research. We asked them to report any snake encounters in the study area and requested that they call us if they came across any snakes. In the study area, there is a tendency for snake to be subject to human intervention. We further collected the associated data like GPS location, date, month, and year of the snake sighting. We also included the observations of road-killed snake specimens. The species sighted were identified using snake identification keys from the standard taxonomic literature^25^.

### Meteorological data

We obtained meteorological data, from January 2019 to May 2022 including monthly averages of temperature, rainfall, and humidity, from the Indian Meteorological Department for the duration of our study.

### GIS analyses

Each recorded individual was identified up to species level, and its geographic location was noted using a Garmin e-Trex H handheld GPS unit.

### Ordinary kriging

We used Ordinary kriging a spatial interpolation approach that considers not only the distance between the snake sightings but also the overall spatial arrangement (i.e. distance and direction) among the sample points or their autocorrelation^26^.

For the extent of the study area and the sample size, we have optimally selected grid size as a 2 km × 2 km regular grid and all individuals present in each grid were counted. Individual snakes were not separated by species, as the goal was to identify the areas having more snakes. The computation was performed using ArcGIS 10.2 Geostatistical Analyst extension (ESRI 2004).

### Average nearest neighbor distance

We used the Geostatistical Analyst extension of ArcGIS to perform the average nearest neighbour distance test analysis to detect the patterns of distribution of 12 snake species found in the study area. This tool is expressed as the ratio of observed to the expected distance between individuals. In a hypothetical random distribution, the expected distance is the mean distance between neighbours. This analysis yields three values: the nearest neighbour index (R-value), the value of z, and the p-value. A clustered distribution has an R-value of one, whereas a dispersed distribution has an R-value greater than one. If the R-value is 1, the default distribution is considered random. A z-value is computed and compared to a critical z for the appropriate N to determine whether or not to reject the null hypothesis, which states that the distribution of individuals of each species within the area is random. If the p-value is 0.01, it is very unlikely that the observed spatial pattern is the result of random processes, and the null hypothesis is rejected. The minimum number of individuals required for analysis is three; thus, the *species Lycodon striatus*, *Oligodon arnensis*, *Echis carinatus* which were also found in urban areas but had fewer than three individuals, were left out of the analysis.

## Results

**Figure 2:**
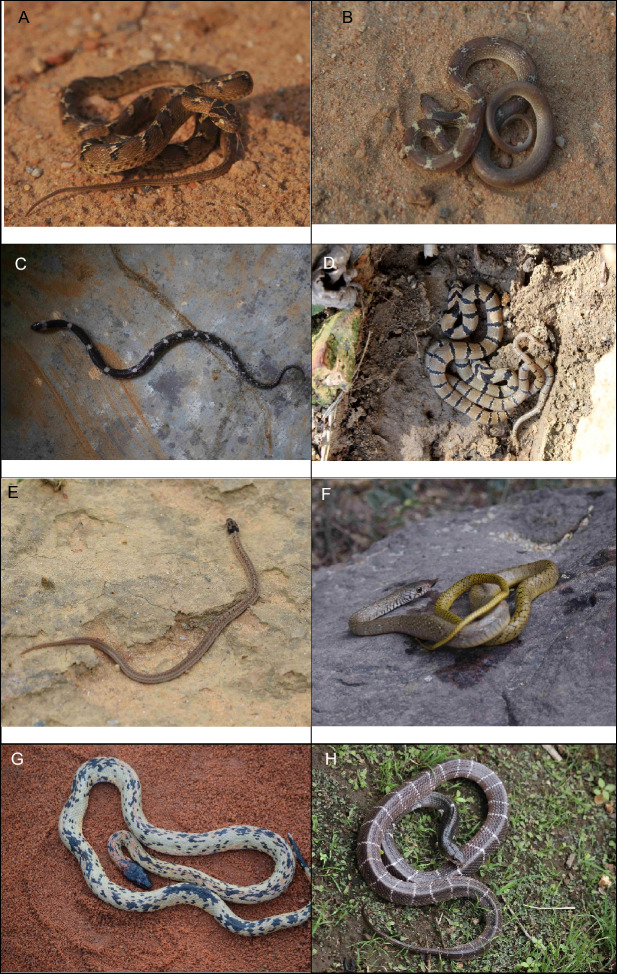

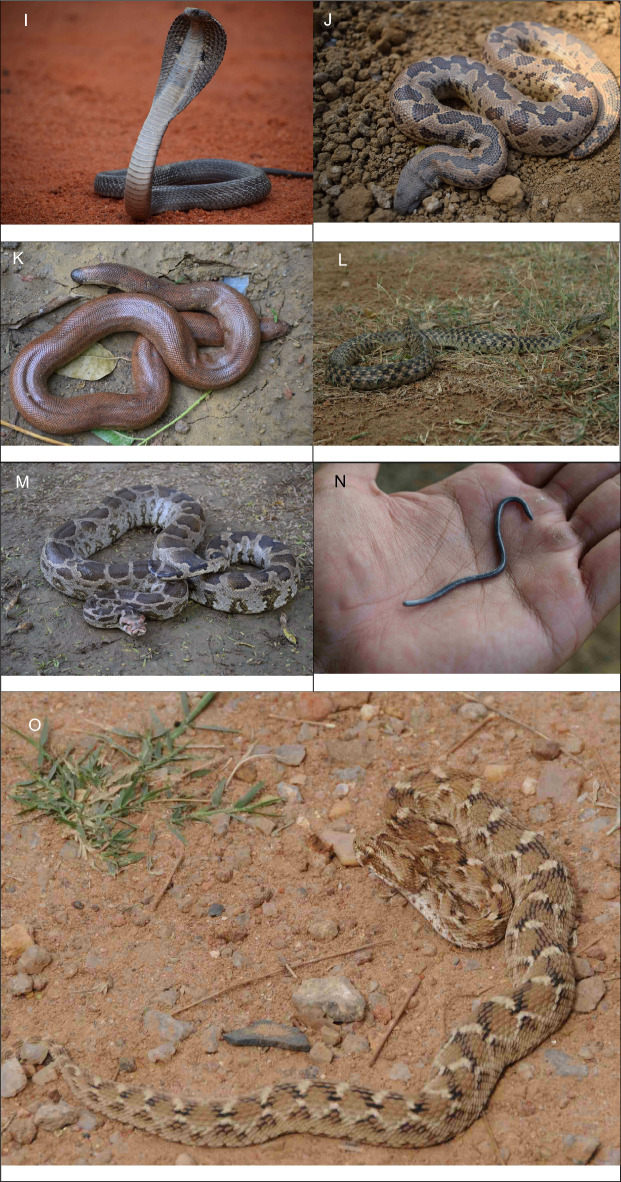
Snake species recorded in the Union Territory of Delhi. (**A**) *Boiga trigonata*; (**B**) *Lycodon aulicus*; (**C**) *Lycodon striatus*; (**D**) *Oligodon arnensis*; (**E**) *Oligodon taeniolatus*; (**F**) *Ptyas mucosa*; (**G**) *Spalerosophis atriceps*; (**H**) *Bungarus caeruleus*; (**I**) Naja naja; (**J**) *Eryx conicus*; (**K**) *Eryx johnii*; (**L**) *Fowlea piscator*; (**M**) *Python molurus*; (**N**) *Indotyphlops braminus*; (**O**) *Echis carinatus.*

Given the paucity of snake sightings, a total of 372 records of snake individuals were recorded being distributed among 15 species, twelve genera and seven families, and (Table 1, Fig. 2A–O) from the union territory of Delhi. Snakes were recorded from wide ranges of habitats like residential streets, rain networks, vacant lots, storerooms of houses, gardens, backyards, cracked walls, prolonged piled-up waste, rock crevices, rivers, forests near wetlands etc.

**Table 1:** Species recorded per species in the Union territory of Delhi between January 2019 and May 2022.

Family	Scientific name	Common name	IUCN status	Indian WPA	n	%
Colubridae	*Boiga trigonata* (Schneider in Bechstein, 1802)	Indian Gamma Snake	LC	IV	4	1.08
	*Lycodon aulicus* (Linnaeus, 1758)	Common Wolf Snake	LC	IV	50	13.44
	*Lycodon striatus* (Shaw, 1802)	Barred Wolf Snake	LC	IV	2	0.54
	*Oligodon arnensis* (Shaw, 1802)	Common Kukri Snake	LC	IV	2	0.54
	*Oligodon taeniolatus* (Jerdon, 1853)	Streaked Kukri Snake	LC	IV	3	0.81
	*Ptyas mucosa* (Linnaeus, 1758)	Oriental Ratsnake	LC	II	139	37.37
	*Spalerosophis atriceps* (Fischer, 1885)	Diadem Snake	LC	IV	18	4.84
Elapidae	*Bungarus caeruleus* (Schneider, 1801)	Common Krait	LC	IV	13	3.49
	*Naja naja* (Linnaeus, 1758)	Spectacled Cobra	LC	II	73	19.62
Erycidae	*Eryx conicus* (Schneider, 1801)	Rough-tailed Sand Boa	NT	IV	14	3.76
	*Eryx johnii* (Russell, 1801)	Red Sand Boa	NT	IV	18	4.84
Natricidae	*Fowlea piscator* (Schneider, 1799)	Chequered Keelback	LC	II	15	4.03
Pythonidae	*Python molurus* (Linnaeus, 1758)	Indian Rock Python	NT	I	10	2.69
Typhlopidae	*Indotyphlops braminus* (Daudin, 1803)	Brahminy Blindsnake	LC	IV	10	2.69
Viperidae	*Echis carinatus* (Schneider, 1801)	Saw-scaled Viper	LC	IV	1	0.27

*n* number of Individuals, IUCN red list status (*LC* least concern, *NT* near threatened), *WPA* Wildlife Protection Act, 1972 schedule.

**Figure 3:**
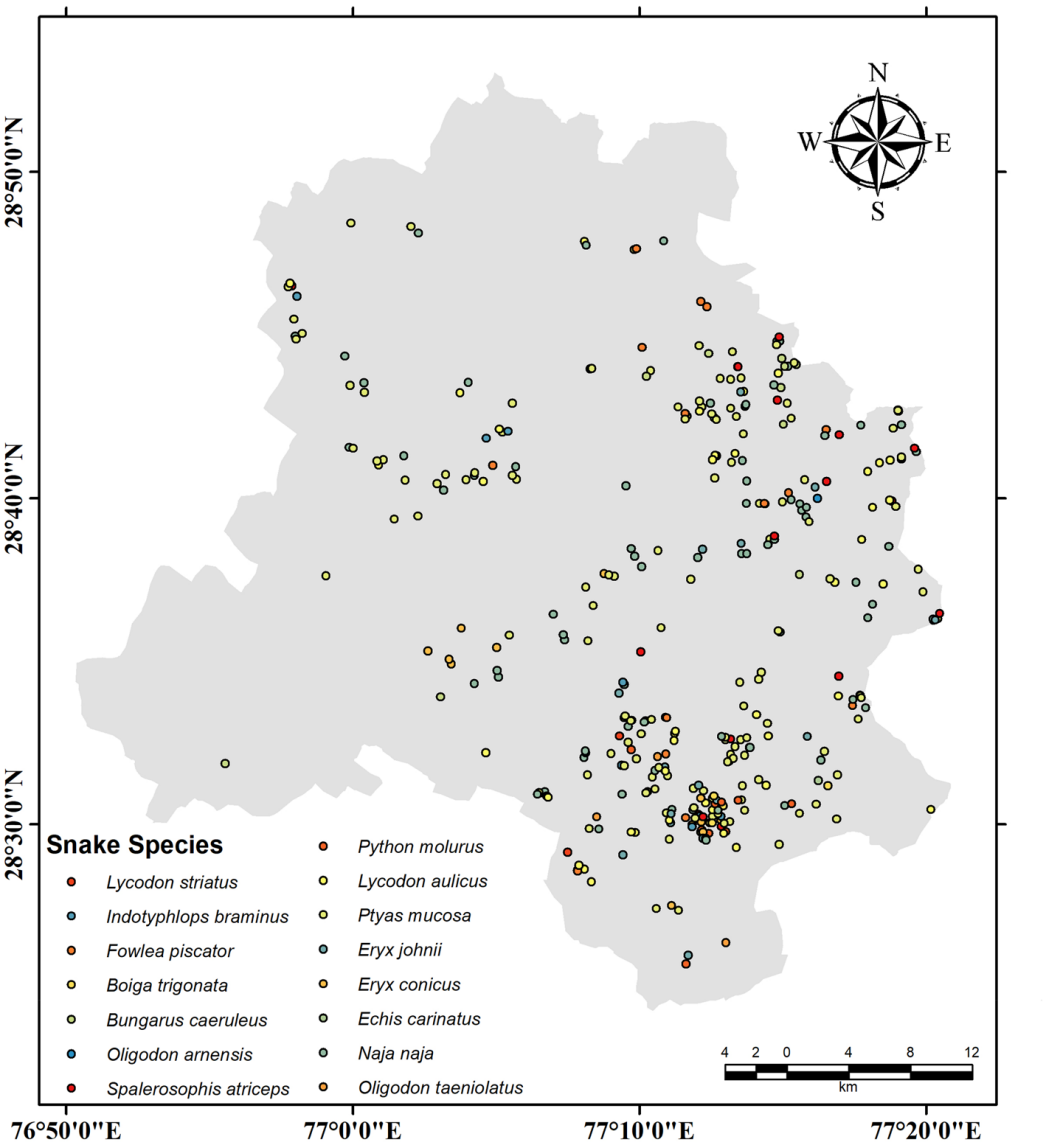
Geographical distribution records of species recorded between January 2019 and May 2022 in the Union Territory of Delhi.

Among the non-venomous species, the most recorded was *Ptyas mucosa* (N = 139) (37.37%) (Fig. 2F) followed by *lycodon aulicus* (N = 50) (13.44%) (Fig. 2B). Within venomous, the *Naja naja* (Spectacled Cobra) (N = 73) (19.62%) (Fig. 2I) was recorded mostly in number. Most species were in the family Colubridae followed by the Elapidae (Table 1). The points of occurrence of snakes are shown in the map (Fig. 3A–O).

**Figure 4:**
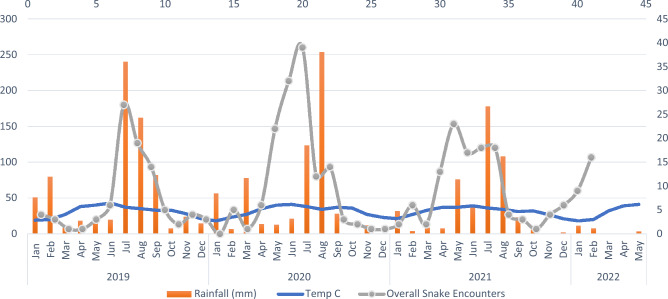
Overall monthly variations in the numbers of snakes recorded with average monthly rainfall and temperature during the study period.

The observation rates were highest in the month of July (22.04%, n = 82) and August (19.89%, n = 74) during the peak monsoon season, showing a defined increasing trend from the month of March and a declining trend after the month of September (Fig. 4).

**Figure 5:**
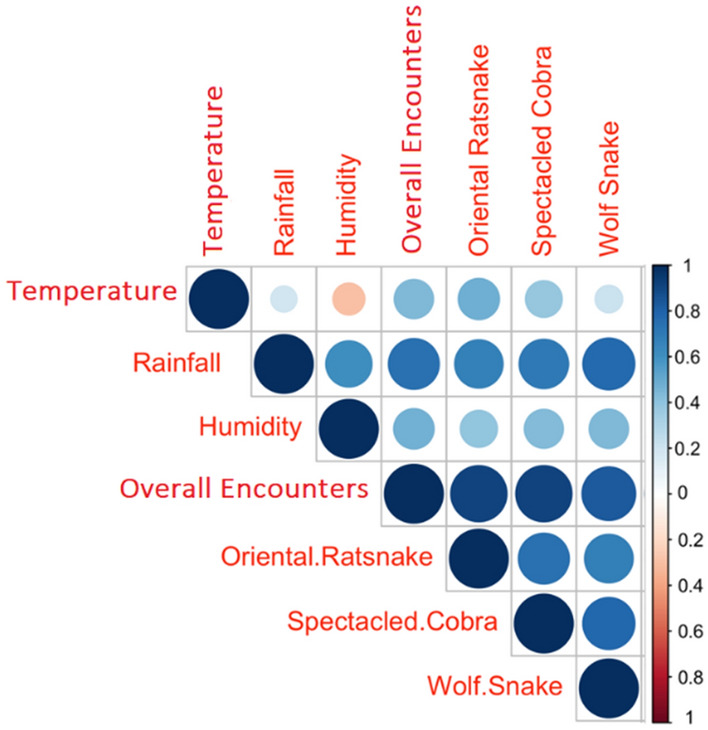
Correlation of different meteorological parameters (temperature, rainfall, and humidity) with overall snake sightings and with sightings of top 3 most common snake species.

**Figure 6:**
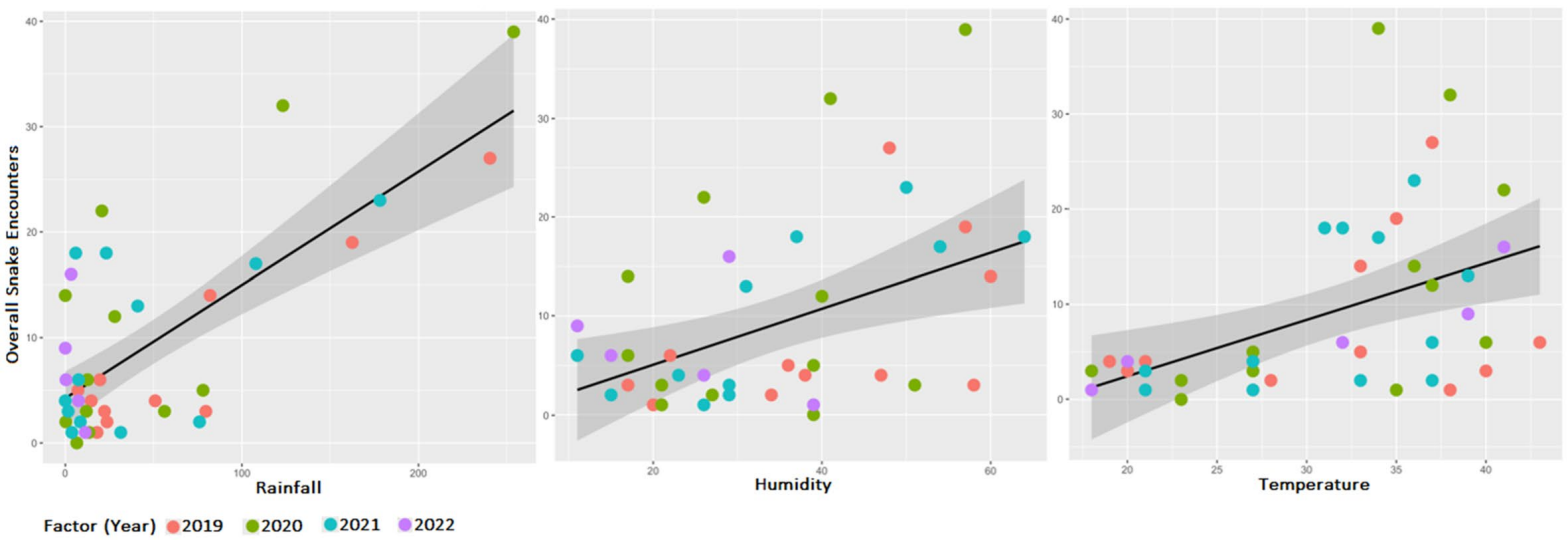
Linear regression analysis of the relationship between monthly rainfall (left), humidity (middle), and temperature (right) with overall snake sightings.

In our analysis from January 2019 to May 2022, a strong positive correlation was observed between total average monthly rainfall and total snake encounters (r = 0.72, R2 = 0.52, p < 0.001, df = 39), and a positive correlation was also found between total snake encounters and total temperature (r = 0.47, R2 = 0.47, p < 0.01, df = 39). This indicates that both rainfall and temperature can predict snake occurrences in diverse land-use configurations (Figs. 5, 6).

Overall snake sightings were explained by monthly meteorological parameters across 41 (Jan 2019 to May 2022) months, the parameters, Rainfall (0.107 ± 0.02, F = 43, R^2^ = 0.5, p < 0.001), Humidity (0.30 ± 0.1, F = 9.23, R^2^ = 0.17, p = 0.004), and Temperature (0.59 ± 0.18, F = 10.95, R^2^ = 0.20, p = 0.002). Although meterological parameters had auto correlation (temperature-rainfall, temperature-humidity, humidity-rainfall), we explored if the response of different functional groups varied with the meterological parameters (Fig. 6).

The significant result of the Average nearest neighbor distance analysis indicates *Lycodon aulicus*, *Naja naja*, and *Ptyas mucosa* have clustered distribution patterns while *Boiga trigonata* and *Oligodon taeniolatus* presented dispersed distribution. Index values of the nearest neighbor (R), z-values, and corresponding p-values for each species are shown in Table 2.

**Table 2:** Spatial distribution of snakes in the union territory of Delhi based on ordinary kriging.

Family	Species	n	R	Z	p	Pattern
Colubridae	*Boiga trigonata*	4	2.79	6.85	** < 0.01**	D
	*Lycodon aulicus*	50	0.79	− 2.83	** < 0.01**	C
	*Oligodon taeniolatus*	3	3.27	7.51	** < 0.01**	D
	*Ptyas mucosa*	139	0.68	− 7.27	** < 0.01**	C
	*Spalerosophis atriceps*	18	1.19	1.52	> 0.1	R
Elapidae	*Bungarus caeruleus*	13	1.18	1.26	> 0.1	R
	*Naja naja*	73	0.85	− 2.5	** < 0.05**	C
Erycidae	*Eryx conicus*	14	1.15	1.22	> 0.1	R
	*Eryx johnii*	18	1.12	0.88	> 0.1	R
Natricidae	*Fowlea piscator*	15	0.99	− 0.08	> 0.1	R
Pythonidae	*Python molurus*	10	1.24	1.46	> 0.1	R
Typhlopidae	*Indotyphlops braminus*	10	1.19	1.19	> 0.1	R

The darker colors represent a higher probability of finding snakes, and the class represents the number of individuals.

*n* number of Samples, *R* nearest neighbour ratio, *Z* critical value of Z score, *p* level of significance, *D* dispersed, *C* clustered, *R* random.

Significant values are in bold.

**Figure 7:**
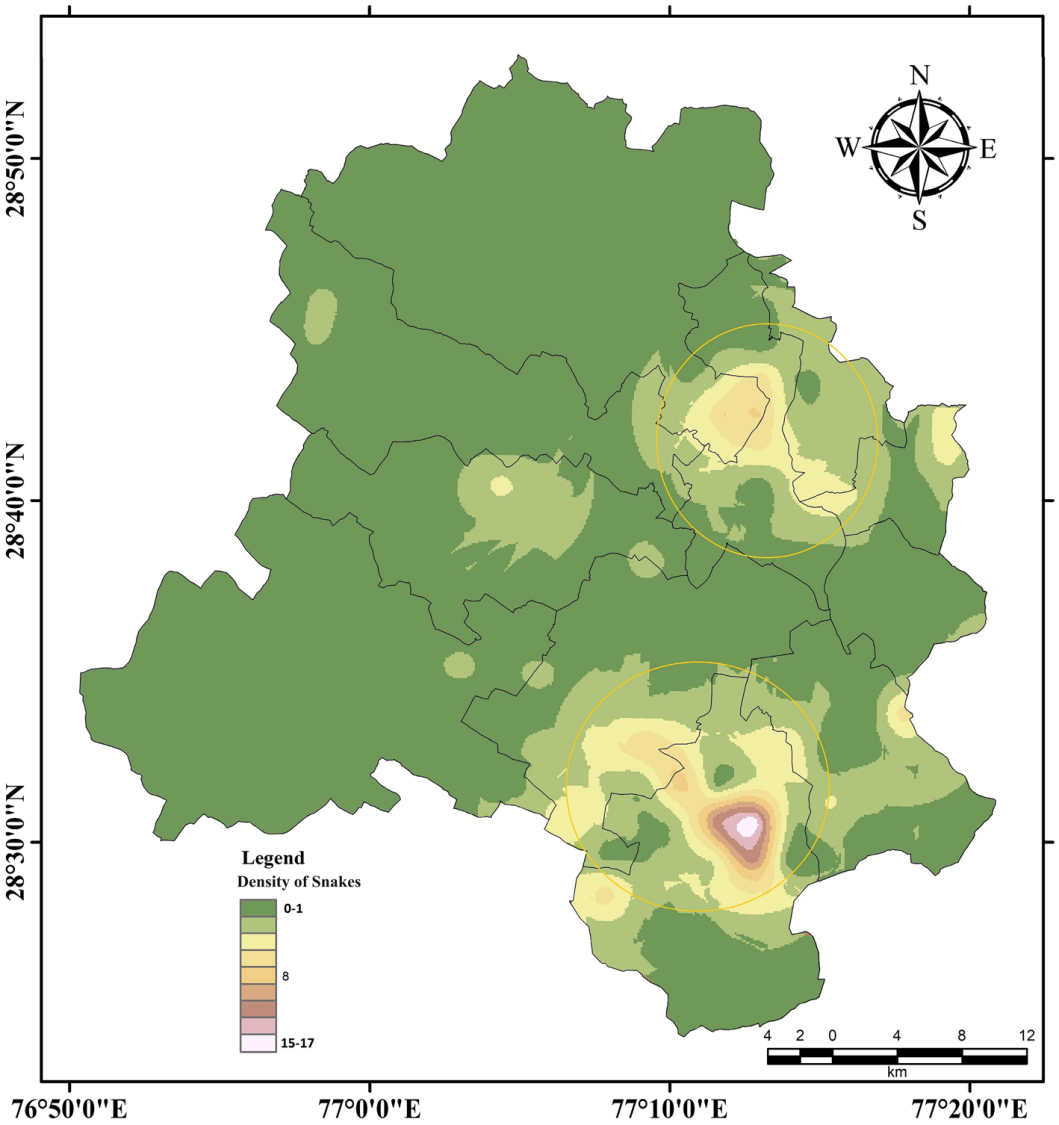
Spatial distribution of density of snakes in Urban Delhi based on ordinary kriging. The two circles represent the areas having a higher probability of finding snakes, and the legend classes represent the Density of individuals. Analysis carried out based on field observations and layout composed using ESRI ArcGIS 10.2 Software.

The population map was prepared using the density of snakes, which ranged from 0 to 17 per grid Ordinary kriging highlighted two distinct areas having concentrated snake encounters in the union territory of Delhi (In circles on the map) The first area in the northeast direction includes Mukherjee Nagar, Wazirabad, Sant Nagar, and Jahangirpuri. The other area in South Delhi comprises Chattarpur, Tughlakabad, Vasant Kunj, Sangam Vihar, Mehrauli, and Ghitorni. Additionally, there were a few other areas with notable snake encounters, including Nangloi, Mundka, and Peeragarhi (Fig. 7).

## Discussion

Throughout the study, the dominant species identified within the study area were *Ptyas mucosa* (Indian Rat Snake), *Naja naja (*Spectacled cobra), and *Lycodon aulicus* (Common Wolf Snake) in Delhi urban setup indicate that these species may have synurbanized, a phenomenon by which animals adapt to urban conditions through ecological and behavioural adaptations^27^. The adaptability to sustain in the wide variety of human-influenced habitats, the predominance of nocturnal habits, and the diet consisting of rodents and invertebrates, which are abundant in urban habitats may be considered a critical factors in the relative abundance of these species, apparently common in the urban environment and thus can be considered “Synanthropic species”.

In the present study, most snake individuals recorded in the union territory of Delhi were non-venomous (76%) and thus do not pose a risk for humans. We didn’t record any snakebite cases throughout our study. Similarly in another study from Australia which is known as the land of venomous snakes, there were no cases of urban snake encounters reported that led to snake bites^28^.

The seasonal abundance of snakes peaked during the monsoon season in Delhi, which corresponds to July to August. Specifically, during the monsoon season, heavy rainfall promotes the abundance of vegetation and the presence of a greater number of small vertebrates like frogs, lizards, toads, and mice, all of which are preferred prey for snakes. This increase in prey availability leads to heightened foraging activity, which can directly influence the number of snake encounters within the city. This highly seasonal activity pattern, as reported in other studies^29,30^, is closely linked to the snakes’ feeding ecology. Moreover, abiotic factors such as rainfall, temperature, and humidity^31^ and biotic factors such as reproduction^32^ are key determinants of snake activity. Low temperatures correspond to reduced metabolism and, consequently, decreased snake activity^32^. From October to January, there is a significant drop in temperature in the study area, which may be one of the important factors responsible for the decline in snake activities.

The significant results for the clustered distribution of snake species *Ptyas mucosa*, *Naja naja* and *Lycodon aulicus* intuitively indicate the generalist diet, their urban adaptations and use of urban and anthropic matrices. These snake species are often found in close proximity to low-income group houses where rat infestations are prevalent, providing an easy and abundant source of food for the snakes, as they are natural predators of rodents. Moreover, the presence of unplastered walls in these houses offers a suitable habitat for house geckos, which, in turn, serve as an additional food source for the snakes^33,34^. Species *Boiga trigonata* and *Oligodon taeniolatus* have selective natural vegetation habitats and non-generalist diets in contrast to *Ptyas mucosa*, *Naja naja* and *Lycodon aulicus* and hence show dispersed distribution^25,33,34^.

We observed the highest number of snake occurrences were in those habitats which were near green spaces and water bodies. Ordinary kriging results highlighted two highly urbanized and populous areas of Delhi (South Delhi district and areas of Northeast and central Delhi Districts), which lies in close proximities of Delhi Ridge and River Yamuna flood plains respectively, which indicates that, due to continuous habitat loss and anthropogenic pressure on the natural environment, snakes have been forced to expand their home ranges into other areas including the urban environment^35^.

Our study also emphasizes the importance of snake rescues and citizen science as a novel way to assess snake patterns in Delhi city, it helped us in achieving large data collection across a wide geographical area in a short period of time which could not be gathered by other means.

Documentation of urban biodiversity is an urgent requirement as the latest statistics, and generated data on urban floral and faunal biodiversity have not been compiled and documented properly. Degradation/fragmentation of habitat, extinction of species, and destruction of unique habitat in urban backdrop need to be monitored. Few snake species recorded from the present study are listed as NT, Near threatened^36^ which indicates the level of threats for these defenseless species. Such inadequacies negatively affect our ability to develop effective conservation actions for these poorly studied species. Thus, to build a better conservation capacity in Delhi, we need to increase the scope of investigations, in reference to habitat loss, reproductive success, factors affecting mortality, and species distributions.

During the study, we observed several threats to the snake population in Delhi, including habitat destruction, habitat fragmentation, negative human perception, lack of awareness, and snake mortality due to vehicular movements. Additionally, our observations revealed that the rescue and release process faces challenges, and there is an urgent need to implement best practices involving minimal handling of snakes. Furthermore, rescue and release activities should be conducted by experienced individuals using scientifically sound methods. These findings underscore the critical importance of preserving Delhi’s unique environment, which comprises the last spurs of the ancient Aravalli mountains, in the form of the Ridge. This region, now fragmented into urban forests or urban parks, holds significant potential to conserve native biodiversity within a densely populated urban area without hindering social and economic development. Nevertheless, the ongoing urban and industrial development across the National Capital Region (NCR) poses a serious threat to herpetofauna habitats and requires immediate attention. Therefore, future research and conservation efforts should focus on utilizing this group of vertebrates as indicator species for habitat management in highly populous cities like Delhi.

## Data Availability

The datasets generated during and analysed during the current study are available from the corresponding author on reasonable request.
